# Profilin-1 suppresses tumorigenicity in pancreatic cancer through regulation of the SIRT3-HIF1α axis

**DOI:** 10.1186/1476-4598-13-187

**Published:** 2014-08-07

**Authors:** Wantong Yao, Shunrong Ji, Yi Qin, Jingxuan Yang, Jin Xu, Bo Zhang, Wenyan Xu, Jiang Liu, Si Shi, Liang Liu, Chen Liu, Jiang Long, Quanxing Ni, Min Li, Xianjun Yu

**Affiliations:** Department of Pancreatic and Hepatobiliary Surgery, Fudan University Shanghai Cancer Center, 270 DongAn Road, Shanghai, 200032 P. R. China; Department of Oncology, Shanghai Medical College, Fudan University, Shanghai, China; Pancreatic Cancer Institute, Fudan University, Shanghai, 200032 P. R. China; The Vivian L. Smith Department of Neurosurgery, The University of Texas Medical School at Houston, Houston, TX 77030 USA

**Keywords:** Pancreatic cancer, Profilin1, SIRT3, HIF1α

## Abstract

**Background:**

Tumor cells exhibit abnormal actin remodeling profiles, which involve the altered expressions of several important actin-binding proteins. Profilin1 (Pfn1), originally identified as an actin-associated protein, has been linked to several human malignancies. Our recent studies suggested that Pfn1 facilitates apoptosis in pancreatic cancer cells. Here, we investigated the exact role of Profilin1 (Pfn1) in pancreatic adenocarcinoma (PDAC) and the underlying mechanisms.

**Methods:**

Pfn1 protein expression in PDAC specimens was analyzed by immunohistochemistry using a tissue microarray (TMA) containing PDAC tumor tissue and corresponding normal tissue samples from 72 patients. The effect of Pfn1 expression on cancer proliferation was assessed in cells by up- and down-regulation of Pfn1 *in vitro* and *in vivo*. Immunoprecipitation and mass spectrometry were used to identify the Pfn1-associated proteins and potential pathways.

**Results:**

Pfn1 was downregulated in clinical pancreatic adenocarcinoma specimens compared with the surrounding benign tissues. Univariate survival analysis of the PDAC cohorts showed that low expression of Pfn1 was significantly correlated with shortened patient survival (mean 14.2 months versus 20.9 months, P < 0.05). Restoration of Pfn1 in pancreatic cancer cells with low endogenous Pfn1 expression resulted in a nontumorigenic phenotype, suggesting that Pfn1 may be a negative regulator of pancreatic cancer progression. Overexpression of Pfn1 *in vivo* decreased the tumor volume in orthotopic xenograft nude mice models. Pfn1 upregulated the expression of SIRT3, leading to HIF1α destabilization. This data revealed that aberrant Pfn1 expression contributes to pancreatic cancer progression.

**Conclusions:**

Our data suggest that Pfn1 is a tumor suppressor in pancreatic cancer that acts via a novel mechanism of regulating the SIRT3-HIF1α axis, independently of its cytoskeleton-related activity.

**Electronic supplementary material:**

The online version of this article (doi:10.1186/1476-4598-13-187) contains supplementary material, which is available to authorized users.

## Background

Pancreatic cancer is a devastating disease, with the highest fatality rate among all cancers and a 5-year overall survival rate of less than 5%. Although some progress has been made in surgery, chemotherapy and radiotherapy in recent decades, the incidence of pancreatic cancer remains equal to its mortality rate. Pancreatic cancer is characterized by uncontrolled growth and rapid progression, and is highly resistant to chemoradiotherapy [1, 2]. Despite advances in determining the biology of pancreatic cancer’s development, little is known about its mechanisms of cell proliferation and signal transduction pathways. Thus, there is an urgent need to understand more about the pathogenesis of pancreatic cancer and to develop new and effective treatments.

The dynamic remodeling of the actin cytoskeleton is involved in multiple cellular functions, such as motility, division, and endocytosis [3, 4]. Moreover, the dynamic equilibrium between monomeric and filamentous actin is altered in neoplastic and/or transformed cells. These actin-remodeling events involve the concerted actions of many different classes of actin-binding proteins (ABPs) [5, 6]. Profilin1 (Pfn1), an indispensable and ubiquitously expressed actin-binding protein, has roles in normal cell motility, proliferation and differentiation [7–11]. Downregulation of Pfn1 expression has been implicated in many types of epithelial-derived tumors, including those originating in the breast, pancreas, liver and bladder [12–15]. Most previous studies focused on investigating Pfn1’s roles and interactions with actin, polyproline ligands and phosphoinositide [16–18]. In addition, a tumor-suppressive action of Pfn1 on breast cancer cell lines was reported, with a novel finding that Pfn1 overexpression was associated with a dramatic upregulation of p27 levels [19]. In this study, we validated Pfn1 as a tumor-suppressor in pancreatic cancer, and identified a novel mechanism of PFN1 regulation of the SIRT3- HIF1α axis, independent of its cytoskeleton-related activity.

## Results

### Expression of Pfn1 is decreased in pancreatic cancer tissues

**Figure 1:**
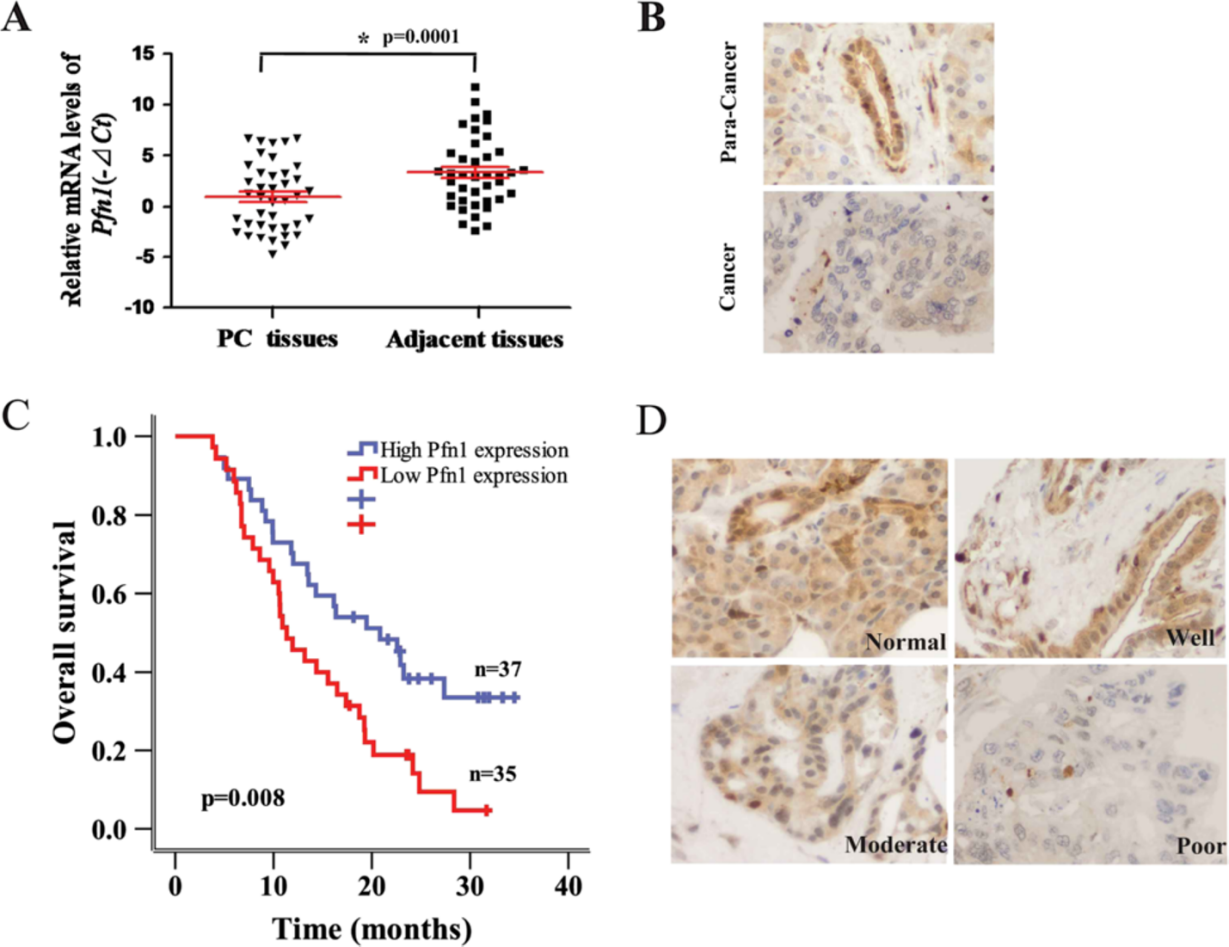
**Downregulation of Pfn1 in pancreatic cancer correlates with poor patient survival. (A)** QPCR analysis of Pfn1 expression in 40 pairs of pancreatic cancer tissues (T) and their corresponding adjacent non-cancerous tissues (ANT). *GAPDH* mRNA expression was used as an internal control. **(B)** Representative micrographs showing low Pfn1 expression in pancreatic cancer and high levels of cytoplasmic expression of Pfn1 in the corresponding adjacent non-cancerous tissues (Magnification, ×400). **(C)** Kaplan-Meier analysis of the correlation between the Pfn1 level and cancer-specific survival of pancreatic cancer patients with high (n = 37) and low (n = 35) Pfn1 expression. **(D)** Representative images of normal, well, moderately, and poorly differentiated tumors (Magnification, ×400).

Quantitative real-time PCR was used to analyze Pfn1 expression profiles of 40 paired pancreatic cancer tissues. Pfn1 expression was downregulated in more than half of the tumors examined (29/40) when compared with their adjacent non-cancerous tissue (p < 0.001, Figure 1A). Immunohistochemistry (IHC) and western blotting were then used to examine the expression of Pfn1 in pancreatic cancer tissues and corresponding adjacent non-cancerous tissues. Consistent with the results for the mRNA levels, Pfn1 expression was downregulated when compared with the corresponding adjacent non-cancerous tissues (Figure 1B and Additional file 1: Figure S1A).

To elucidate the clinical relevance of Pfn1 in pancreatic cancer, we analyzed a cohort of 72 pancreatic cancer specimens using IHC with a Pfn1-specific antibody. Pfn1 is expressed predominantly in the cytoplasm, and two independent pathologists scored its expression in a semi-quantitative manner incorporating both staining intensity and distribution (Additional file 1: Figure S1B). Pfn1 expression levels were strongly associated with cancer-specific survival. Patients with high Pfn1 expression had significant better cancer-specific survival than patients with low Pfn1 expression (Figure 1C). In addition, Pfn1 expression was inversely correlated with tumor differentiation (Figure 1D and Table 1). However, no significant association was found between Pfn1 expression and tumor size, lymph node metastasis or vessel invasion (Table 1, P > 0.05). These data indicated that downregulation of Pfn1 in pancreatic cancer may play a role in cell differentiation, proliferation and progression.

**Table 1:** **Clinicopathological features and correlation with Pfn1 expression in pancreatic ductal adenocarcinoma**

Characteristics	No.	Pfn1-Low Score(-/+)(n = 35)	Pfn1-High Score(++/+++)(n = 37)	P value
Age (y)				0.842^a^
<60	30	15 (20.8%)	15 (20.8%)	
≥60	42	20 (27.8%)	22 (30.6%)	
Gender				0.118^a^
Female	25	9 (12.5%)	16 (22.2%)	
Male	47	26 (36.1%)	21 (29.2%)	
Tumor size (cm)				0.568^a^
<4.0	47	24 (33.3%)	23 (32.0%)	
≥4.0	25	11 (15.3%)	14 (19.4%)	
Tumor differentiation				0.001^a^
Well	13	3 (4.2%)	10 (13.9%)	
Moderate	43	18 (25.0%)	25 (34.7%)	
Poor	16	14 (19.4%)	2 (2.8%)	
Lymph node status (stage)				0.101^a^
Negative (IIA)	44	18 (25.0%)	26 (36.1%)	
Positive (IIB)	28	17 (23.6%)	11 (15.3%)	
Vessel Infiltration				0.068^a^
Negative	56	24 (33.3%)	32 (44.4%)	
Positive	16	11 (15.3%)	5 (7.0%)	
Nerve Infiltration				0.683^a^
Negative	17	9 (12.5%)	8 (11.1%)	
Positive	55	26 (36.1%)	29 (40.3%)	
Median survival (in months)		14.2	20.9	0.008^b^

^a^P values were derived using Pearson chi-square tests.

^b^P values were derived using a log rank test.

All statistical tests are two sided.

*Abbreviations*: *Pfn1* Profilin-1.

### Pfn1 attenuates pancreatic cancer cell proliferation *in vitro*

**Figure 2:**
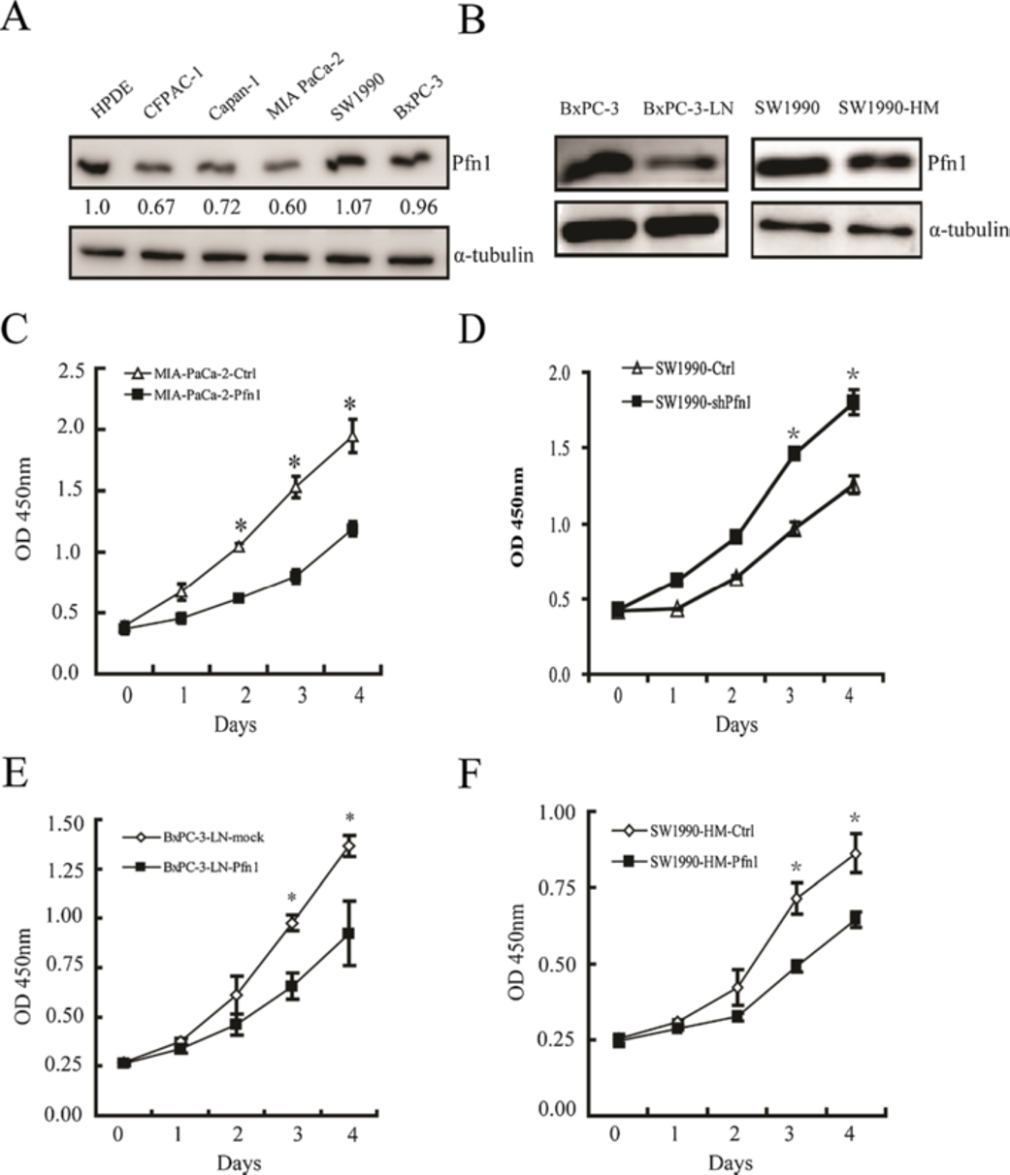
**Overexpression of Pfn1 suppresses pancreatic cancer cell proliferation. (A)**Western blot analysis of Pfn1 expression in five pancreatic cancer cells from the ATCC and human pancreatic ductal epithelium (HPDE) cells. α-tubulin was used as a loading control. **(B)** Pfn1 expression in BxPC-3/BxPC-3-LN and SW1990/SW1990HM cells. **(C-D)** Effect of Pfn1 overexpression and downregulation on the proliferation of MIA PaCa-2 and SW1990 cells. **(E-F)** Effect of Pfn1 overexpression on the proliferation of BxPC-3-LN and SW1990HM cells. (Error bars represent mean ± SD from three independent experiments. *: p < 0.05)

Our clinical findings suggested that Pfn1 might play a role in the tumor differentiation and progression of pancreatic cancer. Therefore, we evaluated the function of Pfn1 in cell proliferation. We selected MIA PaCa-2 cells with relatively low expression of Pfn1 and SW1990 cells with high Pfn1 expression compared with that in human pancreatic ductal epithelium (HPDE) cells (Figure 2A). Cells with Pfn1 overexpression and downregulation were established separately using a lentiviral infection system (Additional file 2: Figure S2A). Cell proliferation assays revealed that overexpression of Pfn1 significantly attenuated pancreatic cancer cell proliferation (P < 0.05, Figure 2C). Conversely, downregulation of Pfn1 promoted proliferation (P < 0.05, Figure 2D). To further evaluate the role of Pfn1 in tumor proliferation, we used two pancreatic cancer cells with high lymph node and liver metastatic properties, BxPC-3-LN and SW1990-HM separately. Compared with their parental BxPC-3 and SW1990 cells, BxPC-3-LN and SW1990-HM expressed lower levels of Pfn1 (Figure 2B). Furthermore, overexpression of Pfn1 also significantly attenuated cell proliferation in these two cell lines (Figure 2E-F; Additional file 2: Figure S2B).

### Pfn1 inhibits pancreatic cancer growth *in vivo*

**Figure 3:**
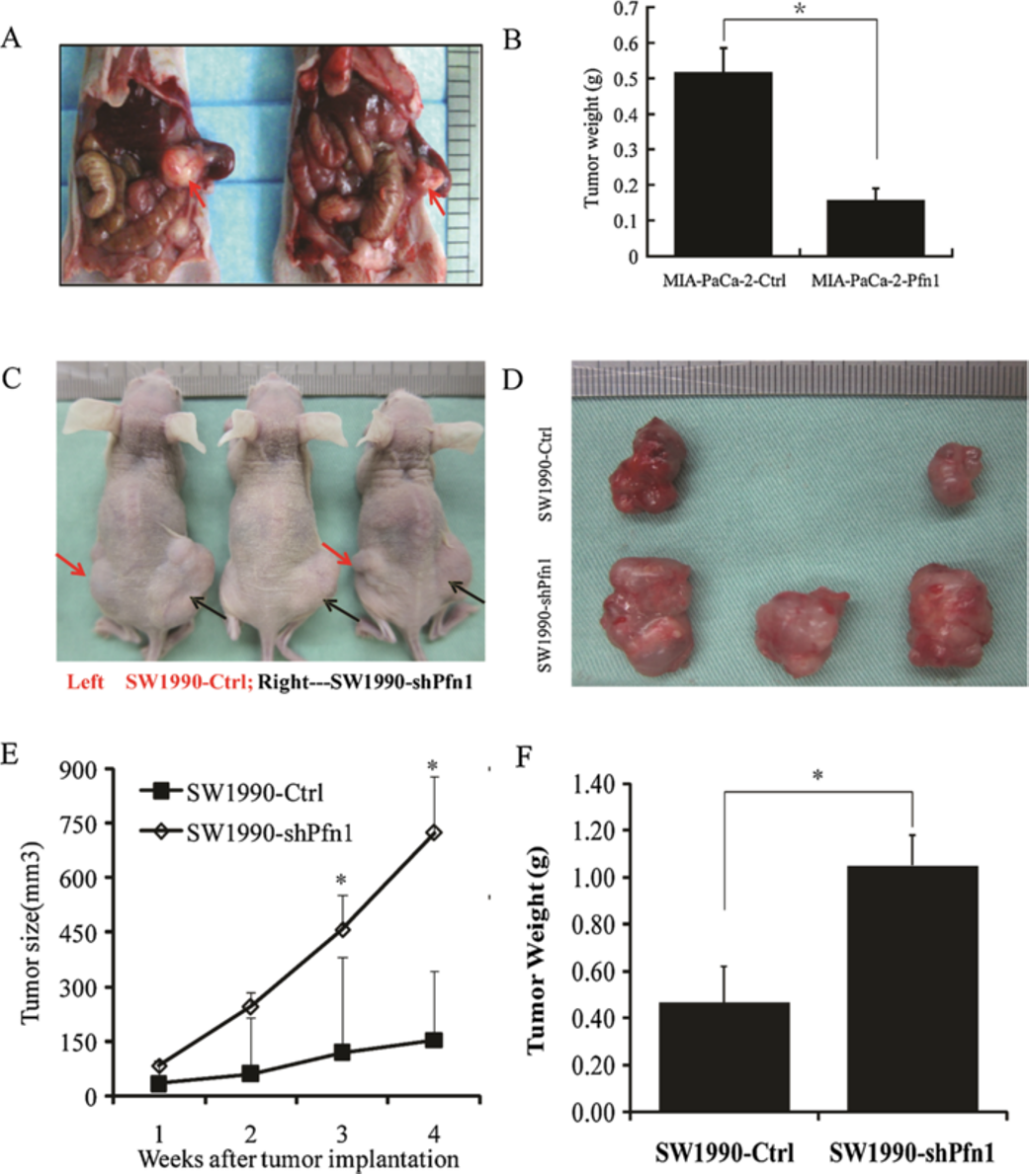
**The effect of Pfn1 expression on tumor growth of pancreatic cancer cell xenografts** ***in vivo*** **. (A)** Representative micrographs of MIA PaCa-2-Ctrl (left) & MIA PaCa-2-Pfn1 (right) orthotopically injected nude mice. **(B)** Each tumor formed by the indicated cells was weighed. **(C-D)** Representative micrographs of SW1990-Ctrl & SW1990-shPfn1 subcutaneously injected nude mice. **(E)** Effect of Pfn1 knockdown (KD) on pancreatic cancer growth. **(F)** Each tumor formed by the indicated cells was weighed. Data are presented as mean ± SD from 5 mice in each group. *: *P* < 0.05.

We next examined the *in vivo* effects of Pfn1 expression on tumor growth. Pancreatic cancer cells were orthotopically injected into the pancreas of nude mice. At the experiment endpoint, the control group had tumors with significantly larger volumes than the Pfn1-overexpressing group (Figure 3A). This was confirmed by the weights of dissected tumors (Figure 3B), strongly suggesting a decrease in tumor cell growth caused by Pfn1 overexpression. Conversely, downregulation of Pfn1 in SW1990 cells promoted tumor growth subcutaneously (Figure 3C-F). The reversal of the Pfn1 overexpression phenotype by Pfn1 knockdown (KD) further demonstrated that Pfn1 exerts a growth-suppressive function in human pancreatic cancer. Accordingly, the level of proliferation related molecules, such as Ki67, PCNA and c-Myc, decreased after Pfn1 overexpression (Additional file 2: Figure S2C).

### Mass spectrometry to identify Pfn1 interacting proteins

**Figure 4:**
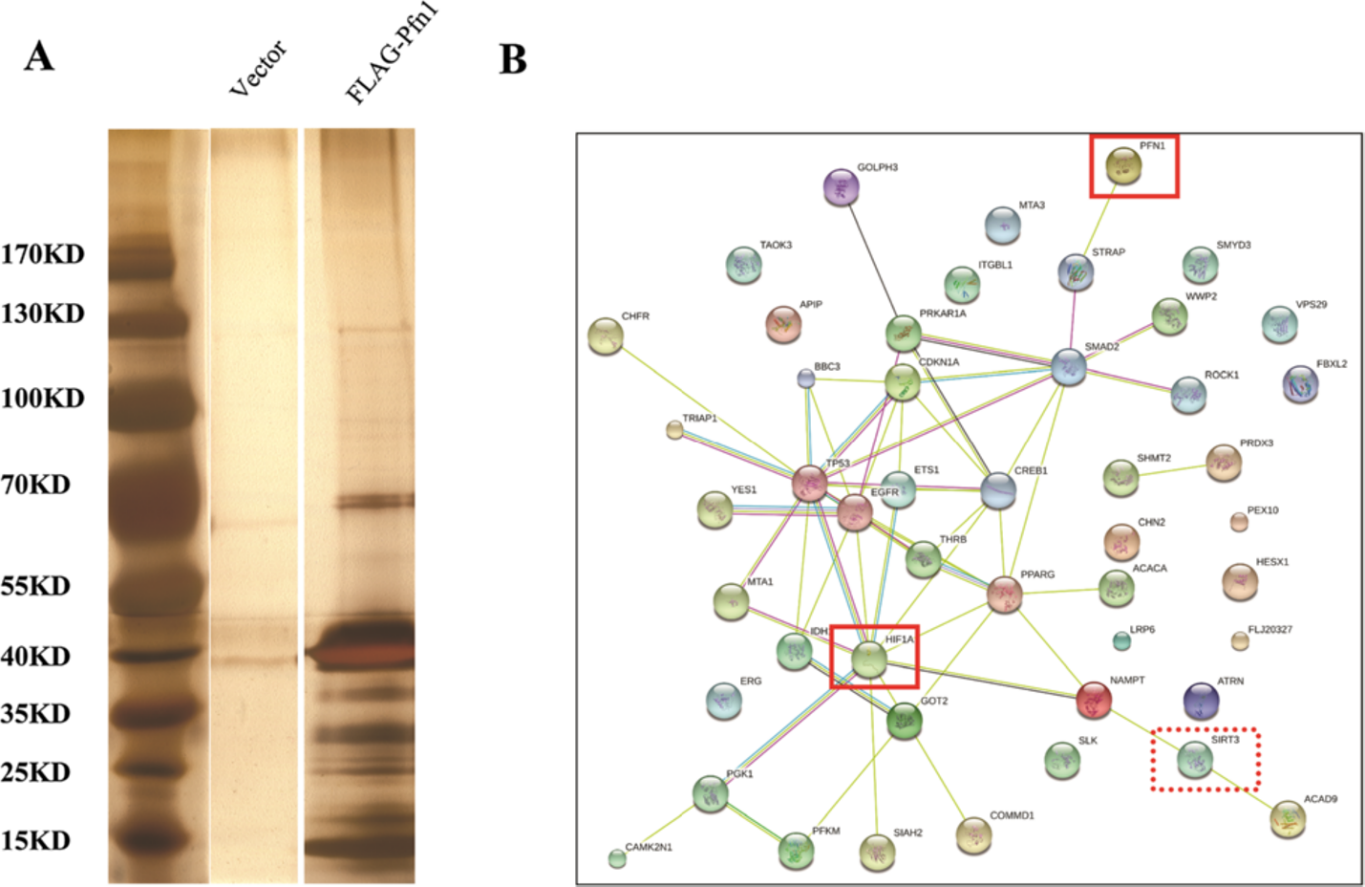
**Identification of Pfn1 interacting protein candidates. (A)** Mass spectrometry analysis of Pfn1-associated proteins. **(B)** Network of the identified Pfn1 interacting partners based on bioinformatic analysis.

In an effort to further understand the physiological functions of Pfn1, we employed immunoprecipitation followed by mass spectrometry to identify the binding proteins. We co-purified a number of proteins that potentially interacted with Pfn1 (Figure 4A). The protein bands were retrieved and subjected to mass spectrometry to identify the candidate proteins. Forty-six proteins were identified (Additional file 3: Table S1). These proteins are involved in diverse cellular functions, such as mitochondrial associated physiological processes, transcriptional control of genes, and endocytosis. Further bioinformatic analysis suggested that Pfn1 might be involved in the process of glycolysis mediated by HIF1α (Figure 4B).

### Pfn1 interacts physiologically with SIRT3

According to our previous study, PFN1 enhanced the apoptosis of pancreatic cancer cells through a mitochondrial pathway [20]. Interestingly, 11 mitochondrial proteins were identified by mass spectrometry (Additional file 3: Table S1). Among them was SIRT3, a sirtuin with NAD-dependent deacetylase activity, which is a tumor suppressor that protects against carcinogenesis by maintaining mitochondrial integrity and efficient oxidative metabolism [21]. Recently, Finley [22, 23] further found that SIRT3 could mediate metabolic reprogramming in human breast cancer cells by destabilizing HIF1α. These observations led us to hypothesize that Pfn1 might, together with SIRT3, regulate HIF1α activity and inhibit tumorigenesis.

**Figure 5:**
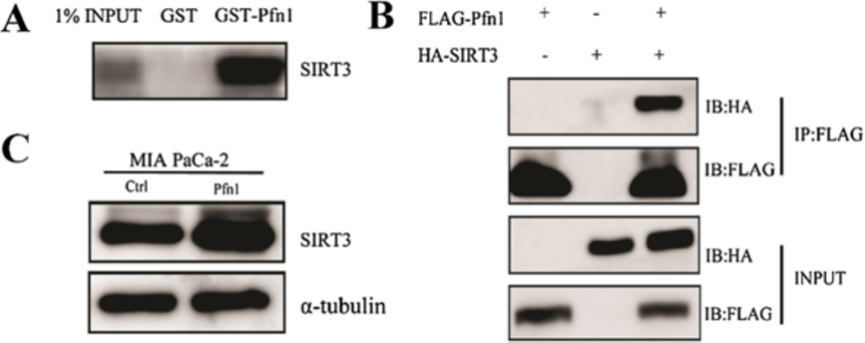
**Pfn1 interacts with SIRT3 directly. (A)** *In vitro* GST-pull-down assay. **(B)** Co-immunoprecipitation assay to verify *in vivo* interaction between Pfn1 and SIRT3. **(C)** Transfection of exogenous Pfn1 resulted in increased SIRT3 expression.

To test this possibility, we first investigated the possibility that Pfn1 interacts with SIRT3 in cancer cells. Using *in vitro* GST pull-down assays, we observed physical interactions between Pfn1 and SIRT3 (Figure 5A). Next, to test the interactions in intact cells, the associations between Pfn1 and SIRT3 were analyzed *in vivo* by coimmunoprecipitation. Pfn1-FLAG and SIRT3-HA fusion constructs were transiently introduced into the cancer cell line MIA PaCa-2. Immunoprecipitation with anti-FLAG M2 affinity beads showed that Pfn1 does associate with SIRT3 *in vivo* (Figure 5B). Moreover, we found that transfection of Pfn1 resulted in an increased SIRT3 protein level (Figure 5C). These results suggested that the interaction between Pfn1 and SIRT3 is specific.

### Pfn1 negatively regulates HIF1α protein levels via SIRT3 directly

**Figure 6:**
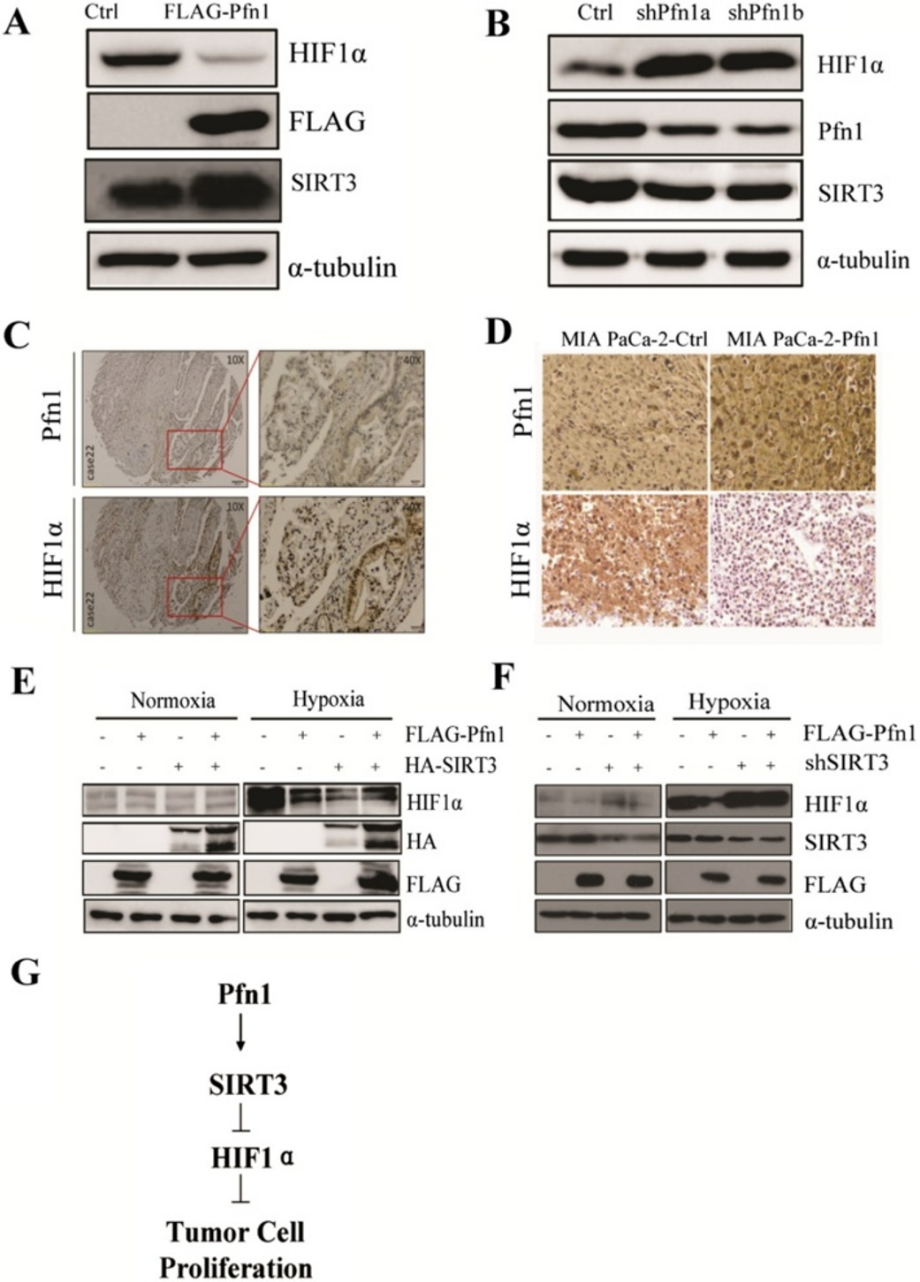
**Pfn1 increases HIF1α protein degradation via SIRT3.** **(A-B)** Effects of Pfn1 overexpression and Pfn1 KD on HIF1α protein level in MIA PaCa-2 cells under hypoxia. **(C)** Immunohistochemical (IHC) staining for Pfn1 and HIF1α in serial sections of clinical samples. **(D)** IHC staining for Pfn1 and HIF1α in slices of implanted tumors formed by the indicated cells (magnification, ×400). **(E)** Effect of transfected Pfn1 and SIRT3, either alone or in combination, on HIF1α levels in HEK-293 T cells under normoxia or hypoxia. **(F)** Effect of transfected Pfn1 and shSIRT3, either alone or in combination, on HIF1α levels in HEK-293 T cells under normoxia or hypoxia. **(G)** Schematic of the regulation of HIF1α by Pfn1*.* Error bars represent mean ± SD from three independent experiments. *: p < 0.05

Firstly, we first performed a dual-luciferase assay to determine whether the HRE promoter activity would change as a result of increasing Pfn1 expression in HEK-293 T cells. Cells were co-transfected with the HRE reporter constructs, pcDNA3-HIF1α, and pcDNA3 as the control. Relative firefly luciferase activities were presented. We observed that overexpressed Pfn1 in HEK-293 T cells inhibited the activation of HRE promoter activity (Additional file 4: Figure S3). Furthermore, under hypoxia, endogenous HIF1α protein levels were downregulated in Pfn1-overexpressed cells. By contrast, in Pfn1-KD cells, HIF1α protein levels increased markedly (Figure 6A-B). However, we did not detect changes in HIF1α mRNA levels in Pfn1 overexpressing MIA PaCa-2 cells, which suggested that Pfn1 exerted a posttranslational effect on HIF1α levels (Additional file 5: Figure S4). We also observed a significant inverse correlation between Pfn1 and HIF1α abundance in clinical samples (Figure 6C). Additionally, we examined HIF1α expression in mouse specimens and found that the proportions and staining signals for HIF1α were substantially lower than those in control tumors, suggesting that the increased HIF1α expression might result from Pfn1 downregulation in pancreatic cancer (Figure 6D).

How Pfn1 couples with SIRT3 to regulate HIF1α remains uncertain. To further explore the mechanism, we transfected Pfn1 and SIRT3, either alone or in combination into HEK-293 T cells. As expected, single transfection of Pfn1 or SIRT3 resulted in decreased HIF1α levels. Strikingly, cotransfection of Pfn1 and SIRT3 had no additive effects, and caused only a modest decrease in the HIF1α level compared with the single transfected control (Figure 6E). Thus, we hypothesized that Pfn1 acts upstream on SIRT3 in the regulation of HIF1α. To test this hypothesis, we examined the influence of SIRT3 loss on the regulation of HIF1α. We inhibited SIRT3 expression using a short hairpin RNA (shRNA) and revealed that dual transfection with Pfn1 and shSIRT3 could not repress HIF1α compared with single transfections of the shSIRT3 construct (Figure 6F). Taken together, these results proved that Pfn1 destabilizes HIF1α via SIRT3.

Multiple reports have documented that HIF1α plays an important role in the glycolytic process characterized by malignant tumors [24–26]. Firstly, the mRNA levels of HIF1α-targeted lactate dehydrogenase A (LDHA) and Glut1 [28] were markedly decreased in Pfn1 overexpressing MIA PaCa-2 cells (Additional file 6: Figure S5A). Furthermore, we measured the expression of HIF1α target genes involved in glycolysis, both in MIA PaCa-2 cells and orthotopic tumor tissues using western blotting and IHC analysis, respectively. Glucose transporter Glut1, which are critical for increased glucose uptake and cell proliferation via aerobic glycolysis in tumorigenesis [27], were reduced in Pfn1 overexpressing MIA PaCa-2 cells and in mice tissues relative to their controls (Additional file 6: Figure S5B-C). Pfn1- overexpressing cells displayed decreased glucose uptake and lactate production (Additional file 6: Figure S5D-E), which are important physiological downstream effects mediated by HIF1α.

Therefore, the data showed that Pfn1 regulates the stabilization of HIF1α via SIRT3. Additionally, the inhibition of crucial HIF1a downstream target genes, which coordinate aerobic glucose consumption, may impair cell proliferation and xenograft growth in pancreatic cancer.

## Discussion

Downregulation of Pfn1 has been previously reported in breast, hepatic, pancreatic, and bladder cancer, in which it contributes to the malignant progression of tumor cells. When overexpressed, it can suppress the proliferation and migration ability of cancer cells. However, the underlying molecular mechanism is not clear. Previous studies of Pfn1 mainly focused on its role in actin polymerization during cell proliferation and migration. In this study, tissue microarray analysis demonstrated that Pfn1 was significantly downregulated in pancreatic cancer, which was in consistent with previous observations from a proteomic study [12]. Furthermore, survival analysis revealed that downregulation of Pfn1 correlated with shorter survival times for pancreatic cancer patients. *In vitro* and *in vivo* experiments showed that loss of Pfn1 expression contributed to cell proliferation and tumor growth. When overexpressed, it significantly suppressed the proliferation of cancer cells. However, the mechanisms underlying this effect of Pfn1 in pancreatic cancer have not yet been delineated. Previously, we showed that up-regulation of Pfn1 could induce apoptosis of pancreatic cancer cells through a mitochondrial pathway [20]. A recent study by our group also demonstrated that overexpression of Pfn1 in pancreatic cancer cells facilitated apoptosis and repressed autophagy induced by irradiation [29]. Multiple studies have demonstrated that mitochondrial metabolism is essential for cell proliferation and tumorigenesis [30, 31]. These results suggest a link between Pfn1 and mitochondria in the tumorigenesis of pancreatic cancer.

To explore the mechanisms underlying the inhibition of pancreatic cancer cell growth mediated by Pfn1, we used mass spectrometry to identify the potential Pfn1-interacting proteins. Eleven mitochondrial proteins were identified, including SIRT3, IDH1, and GOT2. Sirtuins are a conserved family of proteins with NAD-dependent deacetylase and/or mono-ribosyltransferase activity, which regulate numerous cellular processes, including longevity, and various glucose and lipid metabolic pathways. Mammals express seven sirtuins (SIRT1–7). The nuclear sirtuins, SIRT1, SIRT6 and SIRT7, regulate numerous metabolic pathways through deacetylating the key transcription factors, cofactors and regulators. The mitochondrial sirtuins, SIRT3, SIRT4 and SIRT5, regulate the activity of important mitochondrial enzymes to maintain energy homeostasis [32]. Among them, we selected SIRT3 for further investigation because it is a mitochondria-localized tumor suppressor required for the maintenance of mitochondrial integrity and metabolism [21]. As a major mitochondrial deacetylase, SIRT3 also regulates the activity of enzymes that coordinate global shifts in cellular metabolism. SIRT3 promotes the function of the tricarboxylic acid (TCA) cycle and the electron transport chain, and reduces oxidative stress. Loss of SIRT3 triggers oxidative damage and metabolic reprogramming to support proliferation and tumorigenesis [33]. Finley et al. showed that SIRT3 could mediate metabolic reprogramming in human breast cancer cells by destabilizing HIF1α [22]. In the present study, HIF1α was also identified among the protein-protein interaction network of Pfn1, suggesting the existence of a potential cross-talk between Pfn1 and HIF1α stability. To confirm this, under hypoxia, endogenous HIF1α protein levels were downregulated in Pfn1-overexpressed MIA PaCa-2 cells. By contrast, in Pfn1-KD cells, HIF1α protein levels increased markedly. These results prompted us to suggest that Pfn1 might couple with SIRT3 to regulate HIF1α activity and inhibit tumorigenesis in pancreatic cancer.

By GST pull-down and coimmunoprecipitation assays, we confirmed that Pfn1 interacts specifically with SIRT3. Overexpression of Pfn1 resulted in increased SIRT3 protein levels. We also showed that loss of SIRT3 decreased the ability of Pfn1 to destabilize HIF1α, providing direct evidence of Pfn1’s coupling with SIRT3 signaling in the regulation of HIF1α. Future experiments will determine which motif Pfn1 might act to regulate SIRT3 expression.

Pancreatic cancer is characterized by low vascular density and prominent stroma, which is different to most other cancers. This specificity severely affects intratumoral perfusion and causes high levels of hypoxia in pancreatic cancer. In the past decade, it has been revealed that under hypoxic conditions, cancer cells switch their glucose metabolism pathway away from the oxygen-dependent tricarboxylic acid (TCA) cycle to oxygen-independent glycolysis, which is known as “Warberg effect” [34, 35]. This metabolic shift is driven by the hypoxia-inducible factor-1 (HIF1) through transcriptional activation of glycolytic genes and inhibition of those promoting oxidative phosphorylation (OXPHOS) [36].

In our study, one of the most interesting findings was the changes in HIF1α target genes involved in the process of glucose metabolism. The expressions of *GLUT1* and *LDHA* were inhibited remarkably after Pfn1 upregulation, thereby contributing to glycolytic pathways that are crucial for pancreatic cancer progression.

These results highlight how differences in protein expression that leads to distinct outcomes in tumors depend on the expression of collaborating proteins. Thus, the assessment of certain biomarkers for clinical outcomes may require analysis of the interacting proteins that together regulate a biological process. In conclusion, we described a novel regulatory mechanism whereby pancreatic cancer proliferation is inhibited by the interaction of Pfn1 and SIRT3 to destabilize HIF1α, which results in reduced expression of HIF1α target genes that coordinate aerobic glucose consumption.

## Methods

### Cell lines and cell culture

Human pancreatic cancer cell lines MIA PaCa-2, SW1990, BxPC-3, CFPAC-1, and Capan-1 were purchased from American Type Culture Collection. BxPC-3-LN cells with lymph node metastasis properties were established previously in our institute [37]. SW1990-HM cells with liver metastasis properties were a gift from Prof. Meng ZQ [38]. MIA PaCa-2 cells were cultured in Dulbecco's Modified Eagle's Medium (DMEM) supplemented with 10% FBS and 2.5% Horse Serum. SW1990 cells were cultured in L-15 medium supplemented with 10% FBS. BxPC-3 cells were cultured in 1640 medium supplemented with 10% FBS. CFPAC-1 cells were cultured in IMDM medium supplemented with 10% FBS. Capan-1 cells were cultured in IMDM medium supplemented with 20% FBS. The HPDE cell line was cultured in complete keratinocyte serum-free medium supplemented with 50 μg/mL bovine pituitary extract (BPE) and 5 ng/mL epidermal growth factor (EGF) (Gibco/Invitrogen, Rockville, MD, USA). Hypoxia mimetic conditions were chemically generated by treating cells with 200 mM cobalt chloride (CoCl_2_, Sigma, St. Louis, MI, USA) for the indicated times. All cell culture media contained 100 U/mL penicillin and 100 mg/mL streptomycin.

### Tissue specimens

Clinical tissue samples used in this study were diagnosed histopathologically and clinically at Fudan University Shanghai Cancer Center from 2010 to 2012. The histological characterization and clinicopathological staging of the samples were determined according to the current International Union Against Cancer (UICC). For the use of these clinical materials for research purposes, prior patients’ consents and approval from the Institutional Research Ethics Committee were obtained. The samples’ clinical information is presented in Table 1.

### Lentivirus production and host cell infection

To overexpress Pfn1 in pancreatic cancer cells, a lentivirus mediated transfection method was employed. In brief, DNA encoding FLAG tagged Pfn1 was cloned into the lentiviral vector pCDH-CMV-MCS-EF1-puro and pCDH-CMV-MCS-EF1-copGFP (SBI, USA) to generate the pCDH-Pfn1-puro and pCDH-Pfn1-GFP constructs. Lentiviral particles were generated by co-transfection of pCDH-Pfn1 constructs with psPAX2 and pMD2.G into HEK-293 T cells. Pancreatic cancer cells stably expressing FLAG tagged Pfn1 were obtained by lentiviral infection and puromycin screening or flow cytometry sorting.

Cloning vector pLKO.1-TRC (Addgene: 10878) was used to express shRNAs targeting Pfn1 and SIRT3. In brief, oligonucleotides containing 21 bp targeting Pfn1 (5′- GCATGGATCTTCGTACCAAGA -3′) and SIRT3 (5′- CCCAACGTCACTCACTACTTT -3′) were ligated into the pLKO.1-TRC cloning vector to generate the pLKO.1-shPfn1 and pLKO.1-shSIRT3 constructs. pLKO.1-shScr (Addgene:1864) containing a scrambled non-target shRNA was used as the control. To produce lentiviral particles, pLKO.1-shPfn1, pLKO.1-shSIRT3, and pLKO.1-shScr were transfected into HEK-293 T cells with lentiviral packaging vectors psPAX2 and pMD2.G. Lentiviral particles were harvested by collecting media from HEK-293 T cells. Pancreatic cancer cell lines were infected with lentiviral particles, and stable shRNA-expressing cell lines were obtained by screening with puromycin.

### Cell proliferation assay

The Cell Counting Kit-8 (CCK-8; Dojindo) was used to measure cell proliferation, as previously described [39, 40].

### RNA isolation and quantitative real-time PCR

Total RNA was prepared using The TRIzol reagent (Invitrogen, USA). A Takara PrimeScript RT reagent kit was used to obtain cDNA by reverse transcription. Quantitative real-time PCR (qPCR) was used to determine the expression status of candidate genes and GAPDH, using an ABI 7900HT Real-Time PCR system (Applied Biosystems, USA). All reactions were run in triplicate. The primers sets are listed in Additional file 7: Table S2.

### Western blotting

Total proteins (20 μg) were separated by polyacrylamide gel electrophoresis (PAGE) and blotted onto polyvinyl-difluoride membranes (Millipore). After blocking with 5% nonfat milk in PBST at room temperature for 30 minutes, membranes were probed with primary antibodies. Antibodies against Pfn1, SIRT3, HIF1α, and Glut1 were all purchased from Epitomics (USA). FLAG, HA, and α-tubulin antibodies were purchased from Sigma (USA). All secondary antibodies were obtained from Jackson ImmunoResearch (USA). After incubation with primary antibodies, washing with PBST and incubating with secondary antibodies, immunoblots were visualized using the ECL detection kit (Millipore) and a ImageQuant LAS 4000 mini was used to quantify the immunoreactive protein bands.

### Mass spectrometry analysis of Pfn1 associated proteins

Whole cell extracts from HEK-293 T cells transiently expressing FLAG-Pfn1 were subjected to affinity purification with anti-FLAG antibody that was immobilized on agarose beads (Sigma, USA). The purified protein complexes were resolved on SDS-PAGE silver stained, and the bands retrieved for subsequent mass spectrometry.

### Tumorigenesis study

BALB/c-nu mice (5–6 weeks of age, 18-20 g, Shanghai SLAC Laboratory Animal Co., Ltd.) were housed in sterile filter-capped cages. Our Institutional Animal Care and Use Committee approved the animal studies. For intrapancreatic injections, mice were anesthetized with 2.5% sodium phenobarbitol (50 mg/kg i.p.) and a 0.5- to 1-cm incision was made in the left subcostal region. 2 × 10^6^ cells in a volume of 50 μl were injected orthotopically into the body of the pancreas. The peritoneum and skin were closed with a 4.0 surgical suture. A bioluminescent imaging method was used to visualize and monitor the dynamic growth of orthotopically xenografted pancreatic cancer cells. For the subcutaneous (s.c.) tumor model, 5 × 10^6^ cells in 100 μl PBS were injected s.c. into the right or bilateral flanks. Twenty-eight days post-implantation, the mice were euthanized and tumors were surgically dissected. The tumor specimens were weighted and fixed in 4% paraformaldehyde. Samples were then processed for histopathological examination.

### Statistical analysis

All experiments were carried out at least three times. Data were expressed as the mean ± SD, and the differences between any two groups were compared using *t*-tests. The chi-square test was used to analyze the relationship between Pfn1 expression and the clinicopathological characteristics. The survival curve was plotted using the Kaplan-Meier method and compared by using the log-rank test. All statistical analyses were carried out using the SPSS 19.0 statistical software package. P values < 0.05 were considered statistically significant.

## Electronic supplementary material

Additional file 1: Figure S1: **(A)** Western blot analysis of Pfn1 expression in eight pairs of pancreatic cancer tissues (T) and their corresponding adjacent non-cancerous tissues (ANT). α-tubulin was used as a loading control. **(B)** Representative micrographs showing negative, weak, intermediate, and strong positive expressions of Pfn1 in pancreatic cancer tissues and adjacent non-cancerous tissues (Original magnification × 400). (TIFF 1 MB)

Additional file 2: Figure S2: **(A)** Construction of MIA PaCa-2 transfectants stably expressing Pfn1 and SW1990 transfectants with shRNAs against Pfn1 using lentivirus infection. **(B)** Construction of BxPC-3-LN and SW1990-HM transfectants stably expressing Pfn1 using lentivirus infection. **(C)** IHC staining for proliferation-related proteins (Ki67, PCNA, c-Myc) in slices of implanted tumors formed by indicated cells (magnification, ×400). (TIFF 1 MB)

Additional file 3: Table S1: Pfn1 interacting proteins identified by mass spectrometry. (DOC 114 KB)

Additional file 4: Figure S3: A dual-luciferase assay was carried out to determine the influence of increased Pfn1 expression on the HRE promoter in HEK293T cells. (PDF 38 KB)

Additional file 5: Figure S4: QPCR analysis of HIF1α expression in indicated cells. (PDF 51 KB)

Additional file 6: Figure S5: Pfn1 downregulates downstream target genes of HIF1α during glycolysis. **(A)** QPCR analysis of HIF1α downstream target genes during glycolysis. **(B)** Western blot analysis of Glut1 expression in the indicated cells. α-tubulin was used as a loading control. **(C)** IHC staining for Glut1 expression in slices of implanted tumors formed by the indicated cells (magnification, ×400). **(D)** Glucose uptake in Pfn1-WT and -OE MIA PaCa-2 cells. **(E)** Lactate production in Pfn1-WT and -OE MIA PaCa-2 cells. Error bars represent mean ± SD from three independent experiments. *: p < 0.05. (PDF 129 KB)

Additional file 7: Table S2: Sequences of qRT-PCR primers. (DOC 52 KB)
