# Supplementary figures and images for: Profilin-1 suppresses tumorigenicity in pancreatic cancer through regulation of the SIRT3-HIF1α axis
Source: Mol Cancer. 2014 Aug 7;13:187. doi: 10.1186/1476-4598-13-187 (PMC4249601; doi:10.1186/1476-4598-13-187)

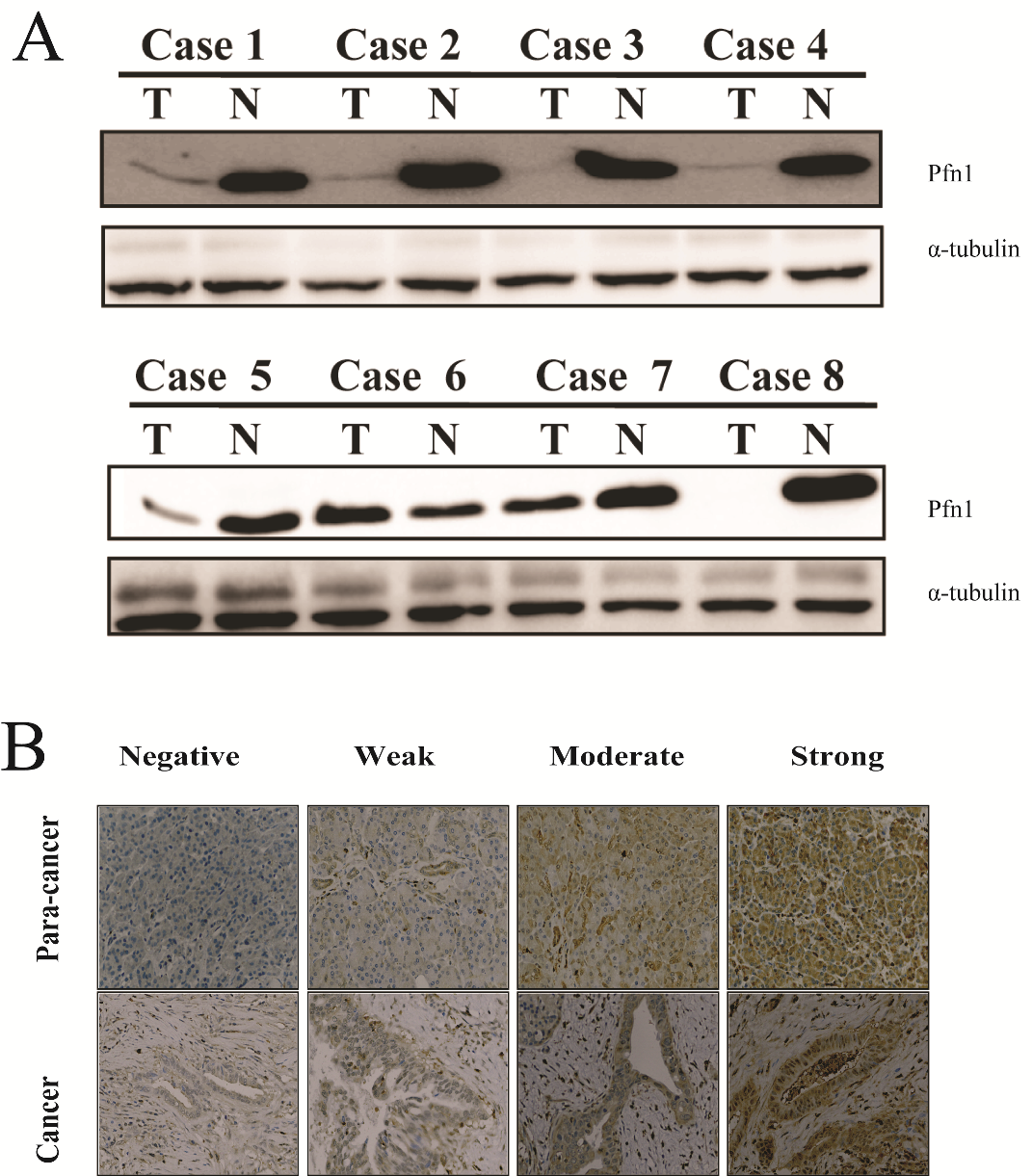

Supplement: Supplementary file 1 — Additional file 1: Figure S1: (A) Western blot analysis of Pfn1 expression in eight pairs of pancreatic cancer tissues (T) and their corresponding adjacent non-cancerous tissues (ANT). α-tubulin was used as a loading control. (B) Representative micrographs showing negative, weak, intermediate, and strong positive expressions of Pfn1 in pancreatic cancer tissues and adjacent non-cancerous tissues (Original magnification × 400). (TIFF 1 MB) [file 12943_2014_1388_MOESM1_ESM.tiff]

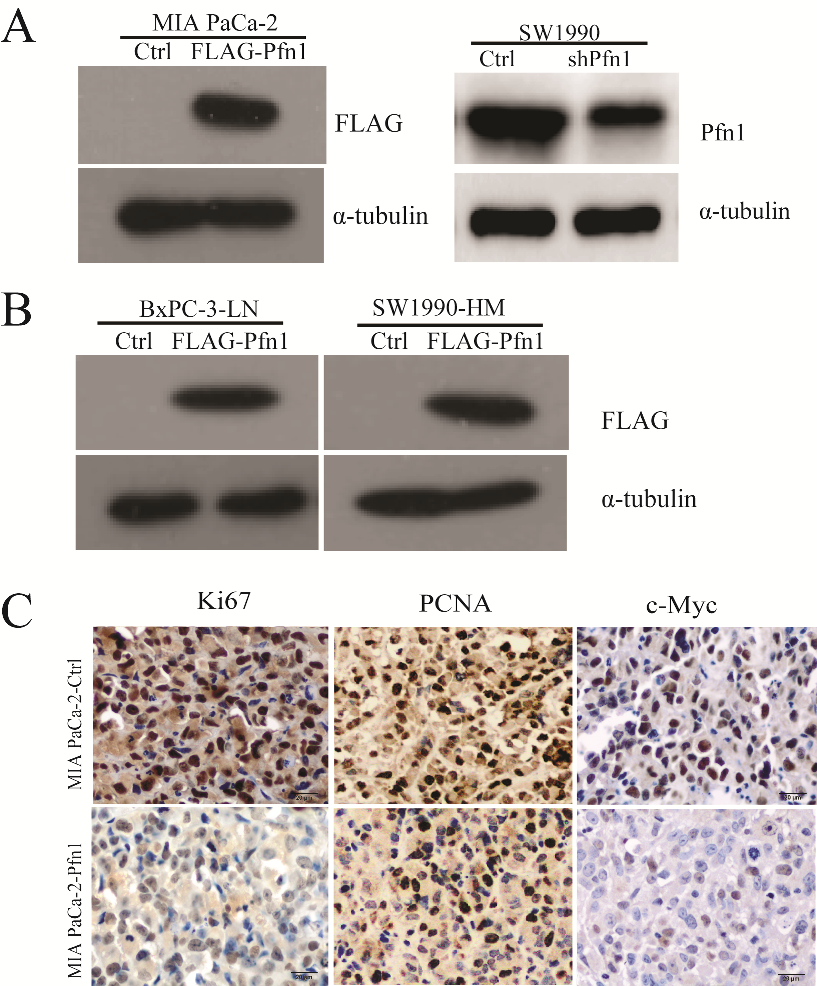

Supplement: Supplementary file 2 — Additional file 2: Figure S2: (A) Construction of MIA PaCa-2 transfectants stably expressing Pfn1 and SW1990 transfectants with shRNAs against Pfn1 using lentivirus infection. (B) Construction of BxPC-3-LN and SW1990-HM transfectants stably expressing Pfn1 using lentivirus infection. (C) IHC staining for proliferation-related proteins (Ki67, PCNA, c-Myc) in slices of implanted tumors formed by indicated cells (magnification, ×400). (TIFF 1 MB) [file 12943_2014_1388_MOESM2_ESM.tiff]

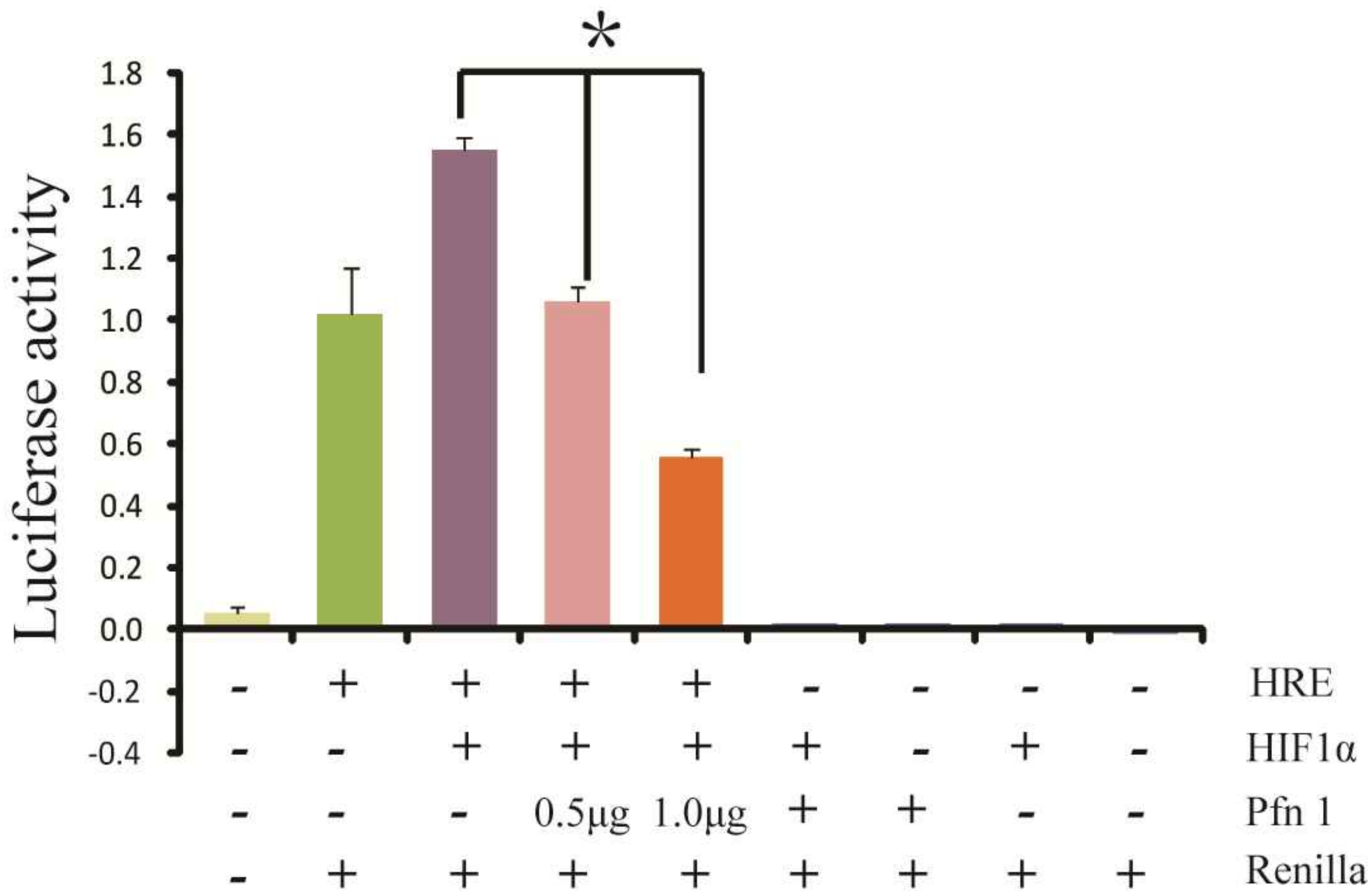

Supplement: Supplementary file 4 — Additional file 4: Figure S3: A dual-luciferase assay was carried out to determine the influence of increased Pfn1 expression on the HRE promoter in HEK293T cells. (PDF 38 KB) [file 12943_2014_1388_MOESM4_ESM.pdf]

Relative expression

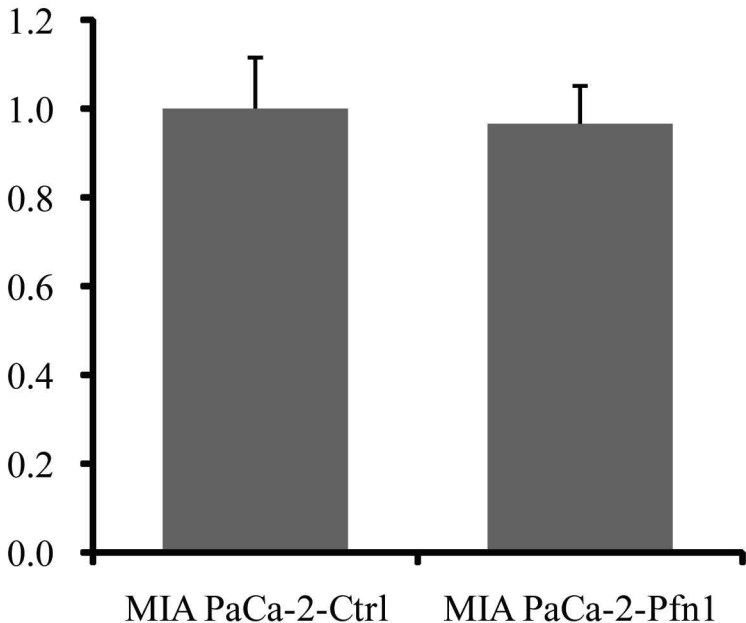

Supplement: Supplementary file 5 — Additional file 5: Figure S4: QPCR analysis of HIF1α expression in indicated cells. (PDF 51 KB) [file 12943_2014_1388_MOESM5_ESM.pdf]

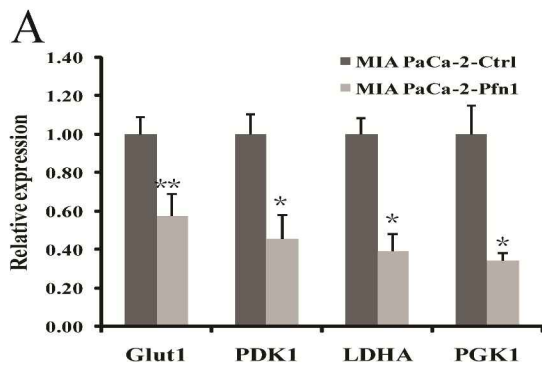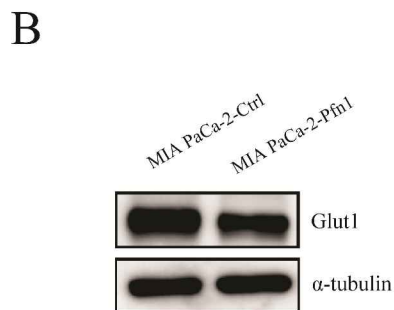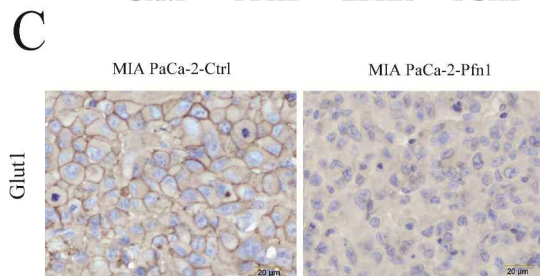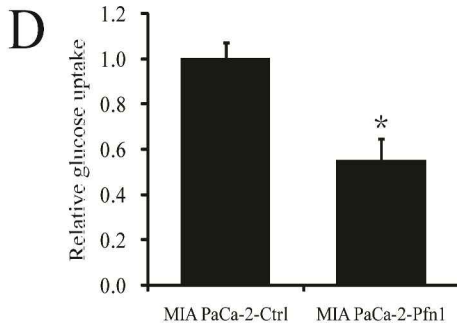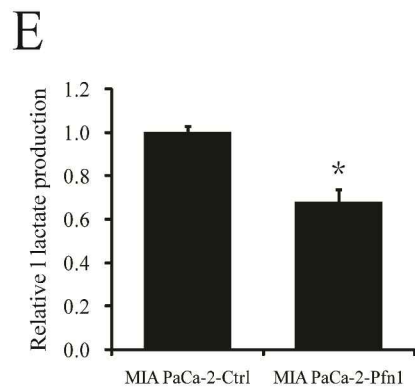

Supplement: Supplementary file 6 — Additional file 6: Figure S5: Pfn1 downregulates downstream target genes of HIF1α during glycolysis. (A) QPCR analysis of HIF1α downstream target genes during glycolysis. (B) Western blot analysis of Glut1 expression in the indicated cells. α-tubulin was used as a loading control. (C) IHC staining for Glut1 expression in slices of implanted tumors formed by the indicated cells (magnification, ×400). (D) Glucose uptake in Pfn1-WT and -OE MIA PaCa-2 cells. (E) Lactate production in Pfn1-WT and -OE MIA PaCa-2 cells. Error bars represent mean ± SD from three independent experiments. *: p < 0.05. (PDF 129 KB) [file 12943_2014_1388_MOESM6_ESM.pdf]
