# Supplementary material for: Profilin-1 suppresses tumorigenicity in pancreatic cancer through regulation of the SIRT3-HIF1α axis
Source: Mol Cancer. 2014 Aug 7;13:187. doi: 10.1186/1476-4598-13-187 (PMC4249601; doi:10.1186/1476-4598-13-187)
Supplement: Supplementary file 3 — Additional file 3: Table S1: Pfn1 interacting proteins identified by mass spectrometry. (DOC 114 KB) [file 12943_2014_1388_MOESM3_ESM.doc]

| **Table S1 Pfn1 interacting proteins identified by mass spectrometry** | | | |
| --- | --- | --- | --- |
| **Protein Name** | **mRNA** | **Protein** | **Localization** |
| ACACA | [NM_198834.1](http://www.ncbi.nlm.nih.gov/nuccore/NM_198834.1) | [NP_942131.1](http://www.ncbi.nlm.nih.gov/protein/NP_942131.1) | cytosol and mitochondiron |
| ZIP4 | [NM_130849.2](http://www.ncbi.nlm.nih.gov/nuccore/NM_130849.2) | [NP_570901.2](http://www.ncbi.nlm.nih.gov/protein/NP_570901.2) | endosome,plasma membrane |
| TAOK3 | [NM_016281.3](http://www.ncbi.nlm.nih.gov/nuccore/NM_016281.3) | [NM_016281.3](http://www.ncbi.nlm.nih.gov/nuccore/NM_016281.3) | plasma membrane and mitochondrion |
| ACTB | [NM_00110.3](http://www.ncbi.nlm.nih.gov/nuccore/NM_016281.3) | [NP_001092.1](http://www.ncbi.nlm.nih.gov/protein/NP_057041.2) | cytoplasm, nucleoplasm, extracellular space, et al |
| APIP | [NM_015957.2](http://www.ncbi.nlm.nih.gov/nuccore/NM_015957.2) | [NP_057041.2](http://www.ncbi.nlm.nih.gov/protein/NP_057041.2) | cytosol |
| VPS29 | [NM_016226.3](http://www.ncbi.nlm.nih.gov/nuccore/NM_016226.3) | [NP_057310.1](http://www.ncbi.nlm.nih.gov/protein/NP_057310.1) | endosome |
| COMMD1 | [NM_152516.2](http://www.ncbi.nlm.nih.gov/nuccore/NM_152516.2) | [NP_689729.1](http://www.ncbi.nlm.nih.gov/protein/NP_689729.1) | nucleus, cytoplasm |
| IDH1 | [NM_005896.2](http://www.ncbi.nlm.nih.gov/nuccore/NM_005896.2) | [NP_005887.2](http://www.ncbi.nlm.nih.gov/protein/NP_005887.2) | cytoplasm, mitochondrion, peroxisome |
| CHN2 | [NM_004067.2](http://www.ncbi.nlm.nih.gov/nuccore/NM_004067.2) | [NP_004058.1](http://www.ncbi.nlm.nih.gov/protein/NP_004058.1) | cytosol ,membrane |
| GOT2 | [NM_002080.2](http://www.ncbi.nlm.nih.gov/nuccore/NM_002080.2) | [NP_002071.2](http://www.ncbi.nlm.nih.gov/nuccore/NM_002080.2) | mitochondrion |
| Thrb | [NM_009380.3](http://www.ncbi.nlm.nih.gov/nuccore/NM_009380.3) | [NP_033406.1](http://www.ncbi.nlm.nih.gov/protein/NP_033406.1) | nucleus |
| LRP6 | [NM_002336.2](http://www.ncbi.nlm.nih.gov/nuccore/NM_002336.2) | [NP_002327.2](http://www.ncbi.nlm.nih.gov/protein/NP_002327.2) | caveola,cell surface,endosome,endoplamic membrane |
| PEX10 | [NM_153818.1](http://www.ncbi.nlm.nih.gov/nuccore/NM_153818.1) | [NP_722540.1](http://www.ncbi.nlm.nih.gov/protein/NP_722540.1) | peroxisome |
| SHMT2 | [NM_005412.5](http://www.ncbi.nlm.nih.gov/nuccore/NM_005412.5) | [NP_005403.2](http://www.ncbi.nlm.nih.gov/protein/NP_005403.2) | mitochondrion |
| ERG | [NM_001136154.1](http://www.ncbi.nlm.nih.gov/nuccore/NM_001136154.1) | [NP_001129626.1](http://www.ncbi.nlm.nih.gov/protein/NP_001129626.1) | cytoplasm,nucleus |
| ROCK1 | [NM_005406.2](http://www.ncbi.nlm.nih.gov/nuccore/NM_005406.2) | [NP_005397.1](http://www.ncbi.nlm.nih.gov/protein/NP_005397.1) | Golgi membrane,cytosol |
| ETS1 | [NM_001143820.1](http://www.ncbi.nlm.nih.gov/nuccore/NM_001143820.1) | [NP_001137292.1](http://www.ncbi.nlm.nih.gov/protein/NP_001137292.1) | nucleus |
| NAMPT | [NM_005746.2](http://www.ncbi.nlm.nih.gov/nuccore/NM_005746.2) | [NP_005737.1](http://www.ncbi.nlm.nih.gov/protein/NP_005737.1) | cytosol |
| ATRN | [NM_139321.2](http://www.ncbi.nlm.nih.gov/nuccore/NM_139321.2) | [NP_647537.1](http://www.ncbi.nlm.nih.gov/protein/NP_647537.1) | cytoplasm |
| SIAH2 | [NM_005067.5](http://www.ncbi.nlm.nih.gov/nuccore/NM_005067.5) | [NP_005058.3](http://www.ncbi.nlm.nih.gov/protein/NP_005058.3) | cytosol,endosome,nucleus |
| CAMK2N1 | [NM_018584.5](http://www.ncbi.nlm.nih.gov/nuccore/NM_018584.5) | [NP_061054.2](http://www.ncbi.nlm.nih.gov/protein/NP_061054.2) | cell junction,plasma membrane |
| TRIAP1 | [NM_016399.2](http://www.ncbi.nlm.nih.gov/nuccore/NM_016399.2) | [NP_057483.1](http://www.ncbi.nlm.nih.gov/protein/NP_057483.1) | mitochondrion,perinuclear region of cytoplasm |
| CHFR | [NM_001161344.1](http://www.ncbi.nlm.nih.gov/nuccore/NM_001161344.1) | [NP_001154816.1](http://www.ncbi.nlm.nih.gov/protein/NP_001154816.1) | PML body,nucleus |
| MTA1 | [NM_004689.3](http://www.ncbi.nlm.nih.gov/nuccore/NM_004689.3) | [NP_004680.2](http://www.ncbi.nlm.nih.gov/protein/NP_004680.2) | nucleus,NuRD complex,endoplasmic reticulum |
| EGFR | [NM_005228.3](http://www.ncbi.nlm.nih.gov/nuccore/NM_005228.3) | [NP_005219.2](http://www.ncbi.nlm.nih.gov/protein/NP_005219.2) | membrane,nucleus |
| PRDX3 | [NM_006793.2](http://www.ncbi.nlm.nih.gov/nuccore/NM_006793.2) | [NP_006784.1](http://www.ncbi.nlm.nih.gov/protein/NP_006784.1) | early endosome,mitochonrion,cytoplasm |
| PFKM | [NM_001166686.1](http://www.ncbi.nlm.nih.gov/nuccore/NM_001166686.1) | [NP_001160158.1](http://www.ncbi.nlm.nih.gov/protein/NP_001160158.1) | cytosol |
| ACAD9 | [NM_014049.4](http://www.ncbi.nlm.nih.gov/nuccore/NM_014049.4) | [NP_054768.2](http://www.ncbi.nlm.nih.gov/protein/NP_054768.2) | mitochondrion,nucleus |
| STRAP | [NM_007178.3](http://www.ncbi.nlm.nih.gov/nuccore/NM_007178.3) | [NP_009109.3](http://www.ncbi.nlm.nih.gov/protein/NP_009109.3) | cytoplasm,mitochondrion,nucleus |
| SLK | [NM_014720.2](http://www.ncbi.nlm.nih.gov/nuccore/NM_014720.2) | [NP_055535.2](http://www.ncbi.nlm.nih.gov/protein/NP_055535.2) | cytoplasm |
| HESX1 | [NM_003865.2](http://www.ncbi.nlm.nih.gov/nuccore/NM_003865.2) | [NP_003856.1](http://www.ncbi.nlm.nih.gov/protein/NP_003856.1) | nucleus |
| GOLPH3 | [NM_022130.3](http://www.ncbi.nlm.nih.gov/nuccore/NM_022130.3) | [NP_071413.1](http://www.ncbi.nlm.nih.gov/protein/NP_071413.1) | Golgi ,endosome,mitochondrion |
| SIRT3 | [NM_012239.5](http://www.ncbi.nlm.nih.gov/nuccore/NM_012239.5) | [NP_036371.1](http://www.ncbi.nlm.nih.gov/protein/NP_036371.1) | membrane,mitochondrion |
| ITGBL1 | [NM_004791.2](http://www.ncbi.nlm.nih.gov/nuccore/NM_004791.2) | [NP_004782.1](http://www.ncbi.nlm.nih.gov/protein/NP_004782.1) | integrin complex |
| PRKAR1A | [NM_001276289.1](http://www.ncbi.nlm.nih.gov/nuccore/NM_001276289.1) | [NP_001263218.1](http://www.ncbi.nlm.nih.gov/protein/NP_001263218.1) | cytosol,plasma membrane |
| MTA3 | [NM_020744.2](http://www.ncbi.nlm.nih.gov/nuccore/NM_020744.2) | [NP_065795.1](http://www.ncbi.nlm.nih.gov/protein/NP_065795.1) | NuRD complex,nucleus |
| FBXL2 | [NM_001171713.1](http://www.ncbi.nlm.nih.gov/nuccore/NM_001171713.1) | [NP_001165184.1](http://www.ncbi.nlm.nih.gov/protein/NP_001165184.1) | cytosol,membrane |
| WWP2 | [NM_001270453.1](http://www.ncbi.nlm.nih.gov/nuccore/NM_001270453.1) | [NP_001257382.1](http://www.ncbi.nlm.nih.gov/protein/NP_001257382.1) | cytosol,nucleus |
| SMAD2 | [NM_001003652.3](http://www.ncbi.nlm.nih.gov/nuccore/NM_001003652.3) | [NP_001003652.1](http://www.ncbi.nlm.nih.gov/protein/NP_001003652.1) | cytoplam,cytosol,nucleoplasm,nucleus |
| PGK1 | [NM_000291.3](http://www.ncbi.nlm.nih.gov/nuccore/NM_000291.3) | [NP_000282.1](http://www.ncbi.nlm.nih.gov/protein/NP_000282.1) | cytosol |
| CREB | [NM_134442.3](http://www.ncbi.nlm.nih.gov/nuccore/NM_134442.3) | [NP_604391.1](http://www.ncbi.nlm.nih.gov/protein/NP_604391.1) | nucleus |
| CDKN1A | [NM_000389.4](http://www.ncbi.nlm.nih.gov/nuccore/NM_000389.4) | [NP_000380.1](http://www.ncbi.nlm.nih.gov/protein/NP_000380.1) | cytosol,nucleus |
| PPARG | [NM_015869.4](http://www.ncbi.nlm.nih.gov/nuccore/NM_015869.4) | [NP_056953.2](http://www.ncbi.nlm.nih.gov/protein/NP_056953.2) | cytosol,nucleus |
| PUMA | [NM_001127241.2](http://www.ncbi.nlm.nih.gov/nuccore/NM_001127241.2) | [NP_001120713.1](http://www.ncbi.nlm.nih.gov/protein/NP_001120713.1) | mitochondrion,cytosol |
| SMYD3 | [NM_001167740.1](http://www.ncbi.nlm.nih.gov/nuccore/NM_001167740.1) | [NP_001161212.1](http://www.ncbi.nlm.nih.gov/protein/NP_001161212.1) | cytoplasm,nucleus |
| YES1 | [NM_005433.3](http://www.ncbi.nlm.nih.gov/nuccore/NM_005433.3) | [NP_005424.1](http://www.ncbi.nlm.nih.gov/protein/NP_005424.1) | cytosol,golgi apparatus |

| **Table S2. Sequences of qRT-PCR primers** | |
| --- | --- |
| Primer | Primer sequence |
| Pfn1-F^a^ | CTCACTGCTGCAGGATGGGGAAT |
| Pfn1-R^b^ | AAAGCTGTGGGGAGCGGTGAA |
| Glut1-F^a^ | CAGTTTGGCTACAACACTGGAG |
| Glut1-R^b^ | GCCCCCAACAGAAAAGATG |
| PGK1-F^a^ | CCACTTGCTGTGCCAAATGGA |
| PGK1-R^b^ | GAAGGACTTTACCTTCCAGGA |
| LDHA-F^a^ | CCCAGTTTCCACCATGAT |
| LDHA-R^b^ | CCCAAAATGCAAGGAACA |
| PDK1-F^a^ | ATTCAAGTTCATGTCACGCTGG |
| PDK1-R^b^ | TTTCCTCAAAGGAACGCCACC |
| HIF1α-F^a^ | GCAAGCCCTGAAAGCG |
| HIF1α-R^b^ | GGCTGTCCGACTTTGA |
| GAPDH-F^a^ | CCACTCCTCCACCTTTGAC |
| GAPDH-R^b^ | ACCCTGTTGCTGTAGCCA |
| ^a^Forward primer | |
| ^b^Reverse primer | |
