# Supplementary material for: Profilin-1 suppresses tumorigenicity in pancreatic cancer through regulation of the SIRT3-HIF1α axis
Source: Mol Cancer. 2014 Aug 7;13:187. doi: 10.1186/1476-4598-13-187 (PMC4249601; doi:10.1186/1476-4598-13-187)
Supplement: Supplementary file 7 — Additional file 7: Table S2: Sequences of qRT-PCR primers. (DOC 52 KB) [file 12943_2014_1388_MOESM7_ESM.doc]

| **Table S2. Sequences of qRT-PCR primers** | |
| --- | --- |
| Primer | Primer sequence |
| Pfn1-F^a^ | CTCACTGCTGCAGGATGGGGAAT |
| Pfn1-R^b^ | AAAGCTGTGGGGAGCGGTGAA |
| Glut1-F^a^ | CAGTTTGGCTACAACACTGGAG |
| Glut1-R^b^ | GCCCCCAACAGAAAAGATG |
| PGK1-F^a^ | CCACTTGCTGTGCCAAATGGA |
| PGK1-R^b^ | GAAGGACTTTACCTTCCAGGA |
| LDHA-F^a^ | CCCAGTTTCCACCATGAT |
| LDHA-R^b^ | CCCAAAATGCAAGGAACA |
| PDK1-F^a^ | ATTCAAGTTCATGTCACGCTGG |
| PDK1-R^b^ | TTTCCTCAAAGGAACGCCACC |
| HIF1α-F^a^ | GCAAGCCCTGAAAGCG |
| HIF1α-R^b^ | GGCTGTCCGACTTTGA |
| GAPDH-F^a^ | CCACTCCTCCACCTTTGAC |
| GAPDH-R^b^ | ACCCTGTTGCTGTAGCCA |
| ^a^Forward primer | |
| ^b^Reverse primer | |
